# Clinical patterns of juvenile idiopathic arthritis in Zambia

**DOI:** 10.1186/1546-0096-11-33

**Published:** 2013-09-14

**Authors:** James Chipeta, Panganani Njobvu, Somwe Wa-Somwe, Chifumbe Chintu, Paul E McGill, Richard Bucala

**Affiliations:** 1 Department of Paediatrics and Child Health, University of Zambia School of Medicine, P.O. Box 50110, Lusaka, Zambia; 2 Department of Paediatrics and Child Health, The School of Medicine and University Teaching Hospital Malaria Research Unit (SMUTH-MRU), D-Block, P/B RW1X, Lusaka, Zambia; 3 Medical Departments of University Teaching Hospital and Maina Soko Military Hospital, Lusaka, Zambia; 4 Department of Rheumatology Stobhill NHS Trust Hospital, Glasgow, Scotland, UK; 5 Department of Medicine/Rheumatology, Yale University School of Medicine, The Anlyan Center, S525, 300 Cedar Street, New Haven, CT 06520-8031, USA

**Keywords:** Juvenile idiopathic arthritis, Zambia, JIA Clinical patterns, HIV-1 seropositivity

## Abstract

**Background:**

Juvenile idiopathic arthritis (JIA) is a heterogeneous group of disorders with different disease manifestations among various populations. There are few reports of JIA among indigenous Africans especially sub-Saharan Africa. We present herein the clinical patterns of JIA encountered at a tertiary hospital in Lusaka, Zambia.

**Method:**

Hospital records of patients with a diagnosis of chronic arthritis with onset at the age of 16 years or less presenting to University Teaching Hospital, Lusaka, Zambia for the periods 1994–98 and 2006–2010 were retrospectively reviewed and reclassified as Juvenile Idiopathic Arthritis (JIA) based on the International League of Associations for Rheumatology (ILA R) JIA diagnostic criteria.

**Results:**

In total, 126 patients with chronic arthritis of onset at age 16 years or less were evaluated over these periods at the hospital. Of these, 85 could further be analyzed by ILAR JIA criteria but 7 (8.24%) were HIV seropositive and were assessed separately. The average age at disease onset among the 78 JIA patients was 8.70 years (range: 1–15 years) with average age at first visit to hospital being 11.3 years (range: 2 to 25 years) and with a female to male ratio of 1.2:1. Polyarticular rheumatoid factor negative JIA, at 34.62%, was the most frequent type of chronic arthritis encountered. Oligoarthritis was found in 32.05% while 11.54% and 14.10% were polyarticular rheumatoid factor positive and systemic JIA, respectively. Enthesitis-related arthritis was found in 6.41% and only 1.28% were determined to have psoriatic arthritis among this population.

**Conclusion:**

JIA is predominantly a polyarticular rheumatoid factor negative disease in Zambia. Late presentation is an issue with major implications for educational input and resource acquisition. There is need to elucidate the genetics and environmental factors of JIA in this region.

## Background

Juvenile Idiopathic Arthritis (JIA) is now known not to be a single disease entity but a heterogeneous group of chronic arthritic disorders of unknown cause in children. Characteristic of these disorders are their onset before the age of 16 years and duration of at least 6 weeks [1]. Reports on the prevalence and incidence of JIA suggest variability among different ethnic and geographically distinct populations [2-5]. Although there are reports from several regions of sub-Saharan Africa, there remains a significant information gap [6-9]. In this paper, we detail the spectrum and epidemiological subtypes of JIA among indigenous black Zambian children seen at a tertiary level hospital. Diagnostic and classification difficulties engendered by limited diagnostic procedures, late presentation to hospital and irregular follow up are highlighted.

## Methods

The records of patients with a diagnosis of arthritis of six weeks or more and of onset at the age of 16 years or less, presenting to University Teaching Hospital (UTH), Lusaka, Zambia during the periods 1994–98 and 2006–2010 were reviewed. UTH is a 1,800-bed tertiary care hospital with a 415-bed Paediatrics and Child Health wing (355 general pediatrics beds and 60 neonatal cots/incubators). There were two data sources. These were out-patient rheumatology clinics at the University Teaching Hospital [UTH] in Lusaka, Zambia from two distinct time periods; a 5-year period from January 1994 to December 1998; a 4-year period from 2006 to 2010. Patients attended these clinics by referral from host hospital doctors for diagnostic evaluation or second opinion on cases that had presented diagnostic difficulties. The patients had been referred by their primary care health facilities within Lusaka and from other parts of Zambia. During 1994–98 patients were evaluated prospectively by PN in an adult rheumatology clinic setting, and by JC from 2006 – 2010 in a new paediatric rheumatology clinic. During the period 2006–2010 we used the ILAR classification [10]. For the period 1994–98 the European Union League of Associations for Rheumatology (EULAR) system was used to classify patients into onset subtypes as follows:

1. Pauciarticular onset (four or fewer joints involved)

2. Polyarticular onset (five or more joints affected)

3. Systemic onset (arthritis associated with spiking fever for at least 2 weeks with or without a typical rash).

This retrospective review of case records involved reclassifying each of the patients by the ILAR diagnostic JIA criteria and then compiling respective clinical data of each patient. Clinical, haematological, immunological, radiological and other relevant findings from the history were obtained from the available records for the review. Anti-nuclear antibody (ANA) tests were not routinely available during either study period and hence it was not part of the analysis. Thus reclassification of each patient into the ILAR subsets was based on the presence of rheumatoid factor, enthesitis and the natural history of the articular disease. Patients were thus re categorized as systemic arthritis, oligoarthritis, polyarthritis (RF negative), Polyarthritis (RF positive), psoriatic arthritis, enthesitis related arthritis (ERA) and others (or undifferentiated). Extended and persistent oligoarthritis ILAR subtypes depend on a period of observation which for many of our patients was not possible due to the retrospective nature of the study, the delay in presentation and the high rates of loss to follow up. Hence in this review we did not sub-classify oligoarthritis into persistent and extended oligoarthritis. Patients who had signs and symptoms of other arthritis such as acute rheumatic fever, septic arthritis, systemic inflammatory disorders (systemic lupus erythematosus, vasculitis, or dermatomyositis), malignancy, human immune deficiency virus type 1 (HIV-1) infection, or metabolic diseases were excluded from the study after careful scrutiny of the respective case records. This study formed part of a general clinical audit of the UTH pediatric rheumatology/immunology clinic of the study period and was approved by the local ethics committee, the University of Zambia School of Medicine Biomedical Research Ethics Committee (UNZABREC).

## Results

### Prevalence of JIA subtypes

**Figure 1:**
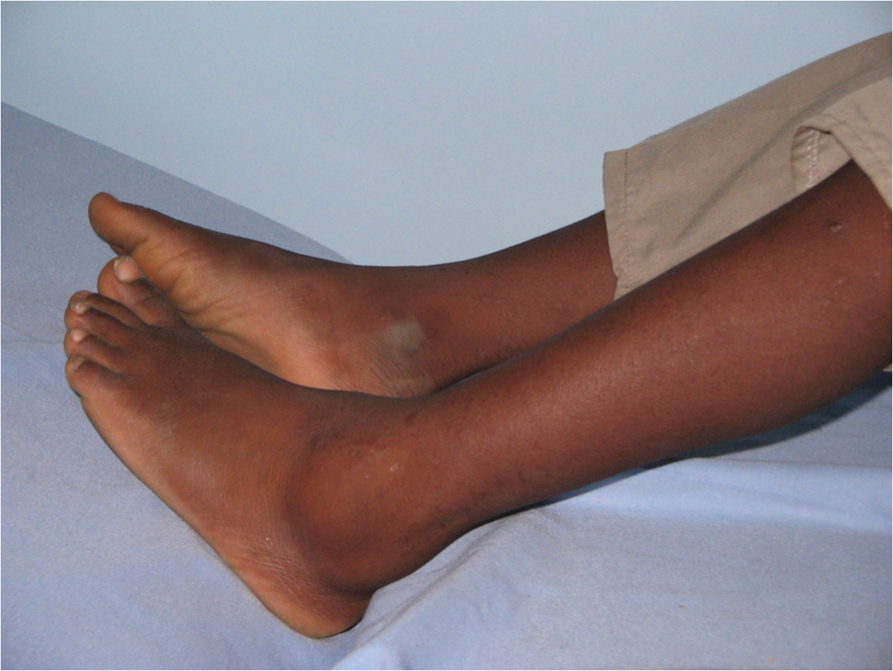
**Ankle joint involvement in Oligoarticular JIA in a Zambian Child.** Photograph showing ankle joint effusion in a Zambian male child with oligoarticular JIA. Note the asymmetrical involvement of the lower big joints. In this case the left ankle joint has effusion while the right is unaffected.

**Figure 2:**
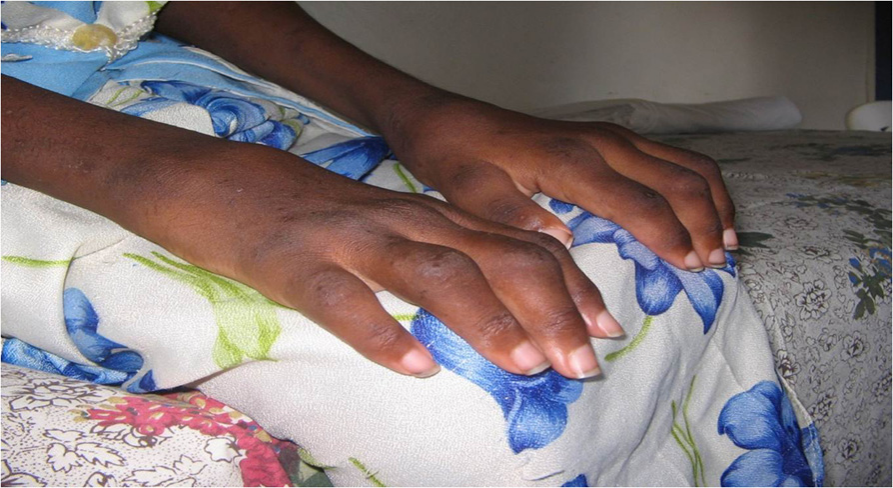
**Rheumatoid factor negative (RF-ve) Polyarticular JIA hand deformities in a Zambian Child.** Photograph showing Juvenile Idiopathic Arthritis (JIA) hand deformities in a female Zambian child with Rheumatoid Factor negative Polyarticular JIA. Note the symmetrical involvement of multiple upper limb joints (In this case the wrist and all the digital < Distal inter-phalangeal, meta-carpophalangeal; Metatarsophalangeal; and proximal inter-phalangeal > joints are involved with multiple deformities).

The JIA subgroup data are shown in Table 1. In total, 126 patients with chronic arthritis of onset at the age of 16 years or younger were seen at UTH. Eight-five of these could further be analyzed by the ILAR JIA criteria. 7 (8.24%) were HIV infected and were excluded from the analysis. The 78 JIA patients comprised 43 girls and 35 boys giving a female to male ratio of 1.2:1. The overall average age at disease onset was 8.7 years (range: 1–15 years) and the majority of the patients presented late with average age at first visit to hospital being 11.3 years (range: 2 to 25 years). Polyarticular rheumatoid factor negative JIA , at 34.6%, was the most frequent type. Oligoarthritis was found in 32.1% while 11.5% and 14.1% were polyarticular rheumatoid factor positive and systemic JIA, respectively. Enthesitis-related arthritis was found in 6.4% and only one child (1.3%) was determined to have psoriatic arthritis among this population. The majority of oligoarticular JIA (M 13 :F 12) had onset in early childhood while a majority of polyarticular JIA (F 17; M 10) had onset in late childhood. Oligoarticular disease affected the lower limbs predominantly (Figure 1). Among patients with polyarticular disease (seronegative and seropositive), the frequency of upper and lower limb involvement was similar and in those with symmetrical arthritis mainly the wrists, MCPs finger PIPs and MTP joints were affected (Figure 2). The pattern of joint involvement at presentation was asymmetric in those with oligoarticular disease and symmetric in polyarticular and systemic onset disease. The sex distribution within subgroups shows a female predominance in RF positive polyarthritis and male predominance in ERA subgroup. The oligoarthritis subgroup had near sex parity and the polyarticular RF negative group had slight female predominance.

**Table 1:** **Profiles of JIA patients presenting at University Teaching Hospital, Lusaka, Zambia from 1994–98** &**2006–2010**

**JIA subtypes**	**Total number (%)**	**Female: male ratio**	**Mean age at disease onset (Years ±STD)**	**Age range at onset (Years)**	**Mean age at presentation (Years ±STD)**	**Age range at presentation (Years)**
Overall	78(100%)	1.2:1	8.70±5.00	1–15	11.3±5.8	2–25
Systemic JIA	11(14.1%)	1:1.2	6±4.3	2–15	8±4.00	4.5–18
Polyarticular RF̄(−) JIA	27(34.6%)	1.7:1	10.70±3.4	3–15	12.89±3.8	4–16
Polyarticular RF̄(+) JIA	9(11.5%)	9:0	11.0±4.5	3–15	16.0±7.0	4–24
Oligoarticular arthritis	25(32.0%)	1:1.1	6.6±4.3	1–14	8.1±4.7	1.6–18
Psoriatic arthritis JIA	1(1.3%)	NA	13	NA	9	NA
Enthesitis related arthritis	5(6.4%)	1:4	10.9±4.8	2–15	17.8±4.2	9–28
Others (undifferentiated)	0(0%)	NA	NA	NA	NA	NA

NB.: *JIA* Juvenile Idiopathic Arthritis, *STD* Standard Deviation, *RF* (−) Rheumatoid Factor negative, *RF*(+) Rheumatoid Factor Positive, *NA* Not applicable.

### Extra-articular clinical features in various JIA subtypes

Fever, still’s rash, eye involvement (acute/chronic uveitis and conjunctivitis), erythrocyte sedimentation rates (ESR) and anemia were the main noted extra-articular clinical features. Hemoglobin (HB) level values and ESR data records were scanty from the available data, the overall erythrocyte sedimentation rates (ESR) were observed to be variable across the various JIA subtypes (Table 2) with an overall average of 52.3 (± 34.4 SD). The overall average HB levels were 11.0 (±2.2 SD) with polyarticular RF (−) showing the lowest HB levels. Ten patients experienced inflammatory eye disease as follows: Chronic uveitis in three with oligoarticular JIA (one blind) and 2 with ERA; Acute uveitis in one each of ERA and polyarticular JIA; Conjunctivitis in one ERA. As expected persistent fever, of at least two weeks duration was observed in all systemic JIA. 37.0% of polyarticular RF(−), 22.2% of polyarticular RF(+) and 16.0% of oligoarticular JIA had episodes of none persistent fever. Still’s rash was identified in one third Systemic JIA subjects.

**Table 2:** Extra-articular clinical features in the various JIA subtypes presenting at University Teaching Hospital, Lusaka, Zambia

**Clinical features**	**Systemic JIA (n = 11)**	**Polyarticular RF(−) JIA (n = 27)**	**Polyarticular RF(+) JIA (n = 9)**	**Oligoarticular (n = 25)**	**Psoriatic arthritis JIA (n = 1)**	**Enthesitis related JIA (n = 5)**	**Overall (n = 78)**
Fever	11 (100%)	10 (37.0%)	2 (22.2%)	4 (16.0%)	1 (100%)	0 (0%)	28 (35.9%)
Still’s Rash	4 (36.4%)	2 (7.4%)	1 (11.1%)	2 (8.0%)	0 (0%)	0 (0%)	9 (11.5%)
Eye Involvement	1 (9.1%)	1 (3.7%)	1 (11.1%)	3 (12.0%)	0 (0%)	4 (80.0%)	10(12.8%)
Acute Uveitis	0 (0%)	1 (3.7%)	1 (11.1%)	0 (0%)	0 (0%)	1 (20.0%)	3 (3.8%)
Chronic Uveitis	1 (9.1%)	0 (0%)	0 (0%)	3 (12.0%)	0 (0%)	2 (40.0%)	6 (7.7%)
Conjuctivitis	0 (0%)	0 (0%)	0 (0%)	0 (0%)	0 (0%)	1 (20.0%)	1 (1.3%)
None	10 (90.9%)	26 (96.3%)	8 (88.9%)	22 (88.0%)	1 (100%)	1 (20.0%)	69(87.2% )
*ESR	40±29.0	59±39.2	53±2.0	44.2±22.3	ND	ND	52.25±34.4
	(n = 3)	(n = 11)	(n = 2)	(n = 5)			(n = 21)
HB	11.1±0.82	10.7±2.47	11.1±0.0	11.56±2.01	ND	ND	11.0±2.2
	(n = 3)	(n = 13)	(n = 2)	(n = 5)			(n = 23)

NB.: *JIA* Juvenile Idiopathic Arthritis, *n* number, *RF (−)* Rheumatoid Factor negative, *RF(+)* Rheumatoid Factor Positive, *ND* Not determined, *ESR* Erythrocyte Sedimentation rate.

*It must be noted that ESR were only available in 21 out of the 78 JIA case series.

### Patient clinical outcomes

The majority of patients in this series (data not shown here) have been lost to follow up. Accordingly, it has been impossible to assess overall clinical outcome mainly due to nonexistence of pediatric rheumatology care prior to the establishment of the current pediatric rheumatology/immunology clinic at UTH. Clinical remission on medication has been documented in 8 patients (3males and 5 females) out of a total 33 JIA patients (24.2%) who are still prospectively being followed by JC in the established pediatric rheumatology/immunology clinic. The average period of remission among these eight patients, to date, is 3.4 ( ± SD 1.1) years. The majority of the documented JIA children in remission, 6 out of the 8 (75%), are with polyarticular RF(−) disease. One of the patients has polyarticular RF(+) disease and the other has oligoarticular JIA. It is worth noting here that, besides facing the challenges of limited basic laboratory diagnostic support services such as RF, ANA, CRP, etc., there is an erratic supply of rheumatology drugs at UTH including basic None-steroidal Anti-Inflammatory Drugs (NSAIDs) and even less availability of common disease modifying anti-rheumatic drugs (DMARDs) such as methotrexate. This limitation results in difficulties in maintaining appropriate JIA treatment protocols at this center and as was the case in this clinical audit affects the objective assessment of long-term patient outcome and disease prognosis.

## Discussion

In the past the heterogeneity in nomenclature of the disease, the lack of diagnostic tests and the differences in diagnostic criteria may have made it difficult to understand fundamental epidemiological comparisons such as incidence, prevalence and clinical manifestations. The current International League of Associations for Rheumatology (ILAR) JIA classification is contributing to a more uniform nomenclature and nosology, and thus improving comparative disease diagnosis and epidemiology across countries and ethnic populations. For this reason we chose to adopt the ILAR classification criteria for this study. We realize that there is an inherent risk of error arising from a retrospective reclassification process (EULAR to ILAR ) for our patients in 1994–98 period prior to the formulation and adoption of the ILAR criteria. However we are confident of the validity of this process because of the meticulous, detailed prospective recordings made by PN and observed by one of us (PEM) during this era. This study covered a total period of 9 years and has yielded 78 cases of JIA. The prevalence of polyarticular sero negative disease (35%) is among the highest reported worldwide, followed by oligoarthritis at 32%, the most common subtype described in studies from Europe and North America [6,7] This finding is in keeping with other studies from the developing world (Table 3) where evidence for a lower incidence of oligoarticular disease has been noted [7-13].

**Table 3:** Comparative JIA Epidemiology: developing and developed countries

**JIA Subtypes**	Zambia*	**South Africa**^**8**^	**India**^**13**^	**Turkey**^**14**^	**Norway**^**11**^	**UK**^**11**^
Number in the studied series	78	78	224	196	172	572
Systemic JIA (%)	14.1	7.7	8	15.3	17.4	14.7
Polyarticular RF (−) JIA (%)	34.6	14	17	30.6	25.8	19.5
Polyarticular RF (+) JIA (%)	11.5	26.9	12	6.6	2.9	5.2
Oligoarticular arthritis (%)	32.1	26	21	34.1	27.3	43.7
Psoriatic arthritis JIA (%)	1.3	1.3	1	1	4.6	7.5
Enthesitis related arthritis (%)	6.4	23	36	10.3	6.3	6.9
Others	0	0	5	2.5	15.6	2
Female : Male Ratio	1.2:1	1:1	-	1:1.1	2.2:1	2.0:1
Eye involvement	11.5	-	-	2	14.5	20
ANA Positivity	-	4.48 (**§**3/67)	-	14.2	41.2	33

*Current Report from Zambia-Chipeta J et al.; **§**Note that ANA was done and available only in 67 of the 78 South African JIA case series. ^8, 13,14,11^ Respective data source references.

About one third of Caucasian children with oligoarthritis which during the first six months affects less than four joints will continue to develop arthritis in more joints thereafter [1]. We did identify prospectively a few who did seem to follow this path. For some of our patients a prolonged delay in presentation to our service and for others failure to return after initial diagnosis (common in our setting) may have masked this dynamic process. It is possible therefore that some children classified as polyarticular may in reality be “oligoarthritis extended”.

The gender ratios in our study show a paucity of preschool girls (especially in the oligoarthritis subset), compared to Caucasian studies where a FM ratio of about 5.1 is the norm. Social and cultural rather than biological reasons may lie behind this observation. It is highly likely that so called “milder” cases of oligoarticular disease might never reach a tertiary care facility in many developing world settings. In the developed world children with oligoarthritis will usually be reviewed in a hospital setting and have access to diagnostic and therapeutic facilities not yet available in most of sub-Saharan Africa. It is apparent that differences in prevalence of oligoarthritis JIA in Africa from those reported in the industrialized west may simply be the result of a selection bias imposed by a dearth pediatric rheumatology services and expertise. In this context it is of interest that in true community based studies in the developing world the prevalence of oligo-articular disease matches or exceeds that of polyarticular disease [14-17].

Our five patients with ERA matched the pattern found world-wide, predominantly a male disease mostly affecting the lower joints with relative late childhood onset. However in Caucasian and Indian studies the majority of subjects with ERA are B27 positive and the prevalence of ERA is much higher than our study and this is likely due to the virtual absence of the B27 gene in our black population [17,18].

An important confounding factor in the assessment of ERA subjects is the ever present risk of HIV infection which is strongly associated with spondyloarthropathy in the African setting including children and juveniles. Enthesitis is a “red flag” signal indicating the need for an HIV test [19,20].

The extra-articular features were as expected, apart from the poor ophthalmic outcomes in those with chronic uveitis. As rheumatology knowledge increases amongst doctors and other care providers in Africa, leading to the application of standard diagnostic and classification criteria, prevalent cases are likely to continue to resemble those reported elsewhere. Clinicians working in parts of Africa where rheumatological services are non-existent or rudimentary face enormous challenges. These include a wide differential diagnostic list and a limited arsenal of diagnostic procedures to aid them in reaching a definitive diagnosis. Limited training in rheumatology, and working in an environment burdened with infectious diseases, their differential diagnostic list is frequently limited to possible infectious causes for rheumatological problems. Distinguishing common Rheumatic Fever from rarer JIA subtypes is one example and unless clinicians are well-trained to recognize the distinctive features of the two conditions, they will have doubts about diagnosing JIA. In our setting oligo JIA may often be attributed to trauma or thought to be infective and children with persistent or more severe joint symptoms may be subjected to arthrotomy and prolonged courses of antibiotics. Therefore, increasing awareness of JIA among clinicians in Africa should lead to improvements in reporting and adherence to the standard diagnostic criteria of the disease.

## Conclusion

All categories of JIA, except the ‘undifferentiated’ (or others), were identified in our Zambian children. Polyarticular RF negative disease was the most common presentation. Young females with oligoarticular disease are underrepresented in this series compared to North American and European series. The subgroup ERA is uncommon and linked to a low prevalence of HLA B27 in the population. Late presentation coupled with the absence of specialized health services are issues with major implications for educational input and resource acquisition.

## Consent

Written informed consent was obtained from the patient for the publication of this report and any accompanying images.

## Abbreviations

JIA: Juvenile idiopathic arthritis; ILAR: International League of Associations for Rheumatology; EULAR: European Union League of Associations for Rheumatology; HIV-1: Human immunodeficiency virus type 1; UK: United Kingdom; UTH: University Teaching Hospital, Lusaka, Zambia; ESR: Erythrocyte sedimentation rate; HB: Haemoglobin; ANA: Anti-nuclear antibody; RF: Rheumatoid factor; HLAB27: Human leukocyte antigen B27; ERA: Enthesitis related arthritis; NSAIDs: Non-steroidal anti-inflammatory Drugs; DMARDs: Disease modifying anti-rheumatic drugs.

## Competing interests

All the authors of this work declare that there are no financial competing interests. This study was undertaken as a part of the ongoing International League of Associations for Rheumatology (ILAR) funded Enhancement of Paediatrics and Adult Rheumatology Education and Practice (EPAREP) project activities at University Teaching Hospital, Lusaka, Zambia.

## Authors’ contribution

JC -Principal Investigator of the EPAREP study and lead author, PN- EPAREP study Co-PI and Contributing author, SS-Contributing author, CC-contributing author, PEM- EPAREP study collaborator and contributing author, and RB-EPAREP study collaborator and contributing author. All authors read and approved the final manuscript.

## Authors’ information

JC-Senior Lecturer and Honorary Consultant Paediatrician, UTH Department of Paediatrics and Child Health; PN-Consultant Rheumatologist, Departments of Internal Medicine of Maina Soko Military Hospital and UTH; SS-Lecturer and Consultant Paediatrician, UTH Department of Paediatrics and Child Health; CC-Professor of Paediatrics and Oncology, UTH Department of Paediatrics and Child Health; PEM-Consultant Rheumatologist, Department of Rheumatology Stobhill NHS Trust hospital, Glasgow, Scotland; RB-Professor of Medicine/Rheumatology, Pathology, Epidemiology & Public Health, Yale University School of Medicine.
